# Body Burdens of Polychlorinated Dibenzo-*p*-dioxins, Dibenzofurans, and Biphenyls and Their Relations to Estrogen Metabolism in Pregnant Women

**DOI:** 10.1289/ehp.8809

**Published:** 2006-01-13

**Authors:** Shu-Li Wang, Yu-Chen Chang, How-Ran Chao, Chien-Ming Li, Lih-Ann Li, Long-Yau Lin, Olaf Päpke

**Affiliations:** 1 Division of Environmental Health and Occupational Medicine, National Health Research Institutes, Miaoli, Taiwan; 2 Graduate Institute of Occupational and Industrial Health, Kaohsiung Medical University, Kaohsiung, Taiwan; 3 Department of Environmental Science and Engineering, National Pingtung University of Science and Technology, Pingtung, Taiwan; 4 Department of Obstetrics and Gynecology, Chung Shan Medical University Hospital, Taichung, Taiwan; 5 ERGO Research Laboratory, Hamburg, Germany

**Keywords:** breast neoplasm, carcinogenic marker, estrogen catabolism, estrogen metabolism, polychlorinated biphenyls, polychlorinated dibenzodioxins, polychlorinated dibenzofurans

## Abstract

Polychlorinated dibenzo-*p*-dioxins (PCDDs, dioxins), polychlorinated dibenzofurans (PCDFs), and polychlorinated biphenyls (PCBs) are environmental endocrine disruptors that have half-lives of 7–10 years in the human body and have toxicities that probably include carcinogenesis. A high ratio of 4-hydroxyl estradiol (4-OH-E_2_) to 2-hydroxyl estradiol (2-OH-E_2_) has been suggested as a potential biomarker for estrogen-dependent neoplasms. In this cohort study of maternal–fetal pairs, we examined the relationship of PCDD/PCDF and PCB exposure to levels of estrogen metabolites in the sera of 50 pregnant women 25–34 years of age from central Taiwan. Maternal blood was collected during the third trimester, and the placenta was collected at delivery. We measured 17 dioxin congeners, 12 dioxin-like PCBs, and 6 indicator PCBs in placenta using gas chromatography coupled with high-resolution mass spectrometry. Estrogen metabolites in maternal serum were analyzed by liquid chromatography tandem mass spectrometry. The ratio of 4-OH-E_2_:2-OH-E_2_ decreased with increasing exposure to 2,3,7,8-tetrachlorodibenzo-*p*-dioxin (β = −0.124, *p* = 0.004 by the general linear regression model, *R* = 0.4). Meanwhile, serum levels of 4-OH-E_2_ increased with increasing concentrations of high-chlorinated PCDFs (i.e., 1,2,3,4,6,7,8-hepta-CDF: β = 0.454, *p* = 0.03, *R* = 0.30). Altered estrogen catabolism might be associated with body burdens of PCDDs/PCDFs. Our study suggests that exposure to PCDDs/PCDFs significantly affects estrogen metabolism. Therefore, PCDD/PCDF exposure must be considered when using the OH-E_2_ ratio as a breast cancer marker.

Polychlorinated dibenzo-*p*-dioxins (PCDDs), polychlorinated dibenzofurans (PCDFs), and polychlorinated biphenyls (PCBs) are environmental endocrine disruptors that have half-lives of 7–10 years in the human body and exhibit adverse effects on development (Kogevinas 2001), endocrine systems (Wang et al. 2005), neural systems (Jacobson and Jacobson 1996, 2002), immunity (Baccarelli et al. 2002), and reproduction (Guo et al. 2000), even at background exposure levels (Brouwer et al. 1999). Women who were accidentally and heavily exposed to a high dose of dioxins in an industrial accident (factory explosion) in Seveso, Italy, developed premenopausal breast cancer at a rate 2-fold higher than controls (Warner et al. 2002). Thus, studies are warranted on the carcinogenetic mechanism(s) of dioxins and dioxin-like compounds (Schecter and Olson 1997; Schwarz and Appel 2005; Warner et al. 2002).

Recent reviews have suggested 2,3,7,8-tetrachlorodibenzo-*p*-dioxin (TCDD) as a group 1 carcinogen (Steenland et al. 2004). However, more epidemiologic evidence is required for an unequivocal classification. The uncertainty is due to inconsistent findings in human studies. Because the actual exposure usually involves multiple congeners, a more inclusive exposure investigation in humans is important.

Estrogen levels have been positively associated with breast cancer risk in a prospective cohort on the island of Guernsey in the English Channel (reviewed by Clemons and Goss 2001). The carcinogenic effect of estrogens may be attributed to the initiation of estrogen metabolism by cytochrome P450 enzymes CYP1B1, CYP1A1, and CYP1A2 (Badawi et al. 2001; Cavalieri et al. 2001; Nebert and Russell 2002; Yager 2000). As shown in Figure 1, dioxins, PCDFs, and some PCBs, can induce CYP1A1, CYP1A2, and CYP1B1 gene expression by serving as aryl hydrocarbon receptor (AhR) agonists (Safe 2001). CYP1A1 and CYP1B1 catalyze hydroxylation of the A-ring of estradiol (E_2_) to form the catechol estrogen 2- or 4-hydroxyl estradiol (2-OH-E_2_ or 4-OH-E_2_, respectively). The quinone metabolites of 4-OH-E_2_ can interact with DNA and, in turn, lead to depurination of DNA—a potential mutagenesis event (Cavalieri et al. 2001). Indeed, a high 4-OH-E_2_:2-OH-E_2_ ratio has been suggested as a marker for breast neoplasm (Liehr 1999). Incubation of human mammary epithelial MCF-7 and MCF-10A cells with dioxins resulted in a concentration-dependent decrease in the ratio of 4-methoxy-E_2_ (4-MeO-E_2_) to 2-methoxy-E_2_ (2-MeO-E_2_) as an indication of decreased 4-OH-E_2_:2-OH-E_2_ (van Duursen et al. 2003). However, no comparable human studies have been reported to date.

In Taiwan, the onset of breast cancer tends to occur at a younger age than in Western countries, and young patients show poorer prognostic features than their older counterparts (Cheng et al. 2000). New biologic markers to identify high-risk groups are urgently needed so that the young high-risk patients can receive treatment as early as possible. In premenopausal women, the levels of sex steroid hormones change dramatically during the menstrual cycle. Thus, it is very difficult to correlate the alterations of hormone status with exposure of environmental endocrine disruptors. In contrast, concentrations of steroid hormones are much more stable during the third trimester of gestation. The present study is aimed to determine estrogen metabolites in maternal blood collected at the third trimester and to examine a possible correlation of the metabolite profile with placental levels of PCDDs/PCDFs and PCBs. This may help answer the question of whether 4-OH-E_2_:2-OH-E_2_ is a good marker to identify the group at high risk for breast cancer in populations exposed to dioxins and dioxin-like compounds.

## Materials and Methods

### Study population and materials.

The study population was described previously (Chao et al. 2004; Wang et al. 2004). In brief, subjects were healthy pregnant women from the general population who were recruited in a medical center located in a suburban setting of central Taiwan. Women (*n* = 763) were recruited between December 2000 and November 2001. A research nurse collected interview data at obstetric clinics during routine health checkups. All of the participants completed questionnaires concerning maternal age, occupation, disease history, cigarette smoking, alcohol consumption, dietary habits, and baby’s stature. Of those recruited, 610 women were ultimately enrolled in the study. Among these, 430 completed the questionnaire and their placentas were collected, and 250 participants provided sufficient maternal venous blood for the chemical analyses. Placental samples and maternal blood samples were analyzed from 50 randomly selected individuals in this group. The placental samples were analyzed for PCDDs/PCDFs and PCBs, and the blood samples were analyzed for estrogens and their metabolites.

The study protocol was reviewed by the Human Ethics Committee of the National Health Research Institutes in Taiwan. We followed the code of ethics established by the Helsinki Declaration of 1964 and revised in 2000 (World Medical Association 2000). Each participant provided informed consent after receiving a detailed explanation of the study and potential consequences.

Maternal venous serum was collected at weeks 28–32 of gestation. Placental samples were collected at delivery. The delivered placenta was cleaned and rinsed with normal saline in the clinic ward. Placental samples were frozen (−20°C) as soon as possible and during transport to the central laboratory in the National Health Research Institutes. At the laboratory, each placenta was divided into four equal parametric parts. One of the quarters was minced and put in sterile Pyrex glass bottles equipped with screw-on caps and Teflon seals provided by ERGO Research Laboratory (Hamburg, Germany). The placental samples, with an average weight of 100 g, were shipped frozen to the ERGO World Health Organization (WHO)-certified laboratory for analysis. This laboratory regularly and successfully participates in interlaboratory comparison studies, including studies of PCDDs/PCDFs in beef and fish liver [National Institute of Public Health (NIPH) 2001].

### Analyses of PCDDs/PCDFs and PCBs.

Analyses of PCDDs/PCDFs and PCBs were performed according to a previously published method (Päpke et al. 1998). Briefly, 100 g of placental sample was extracted with *n*-pentane after addition of an internal standard (^13^C_12_-PCDD/PCDF or ^13^C_12_-PCB). The lipid content of breast milk samples was determined gravimetrically before cleanup in a multi-column system. The specific congeners of 17 2,3,7,8-substituted PCDDs/PCDFs, 12 dioxin-like PCBs (including non-*ortho* and mono-*ortho* PCBs), and six indicator PCBs (International Union of Pure and Applied Chemistry PCB congeners 138, 153, and 180) were analyzed by gas chromatography with high-resolution mass spectrometry (HP GC5890 Series II/VG-AutoSpec; Hewlett Packard, Bristol, UK). Authentic standards of native dioxin-like PCBs and PCDDs/PCDFs were purchased from AkkuStandard Inc. (New Haven, CT, USA). Indicator PCB standards were obtained from LGC Promochem (Wesel, Germany). Two isotope masses were measured for each component. Quantification was performed using internal/external standard mixtures via the isotope dilution method.

The limit of detection (LOD) was defined as the value exceeding the signal-to-noise ratio by a factor of 3. The limit of quantification was defined as 2 × LOD. For each block of samples, individual blanks and laboratory in-house quality control pools for the various matrices were analyzed. The SD was 11–15%. Pools were checked by interlaboratory comparisons. Recovery of ^13^C-labeled internal standards ranged from 70 to 130%. The toxic equivalents (TEQs) of PCDDs/PCDFs and PCBs were calculated according to WHO toxic equivalent factors (Van den Berg et al. 1998).

### Analysis of estrogen and its metabolites.

Each maternal venous serum sample was hydrolyzed with β-glucuronidase and sulfatase at 37°C for 16 hr (Mitamura et al. 2000). Type H-2 β-glucuronidase/sulfatase from *Helix promatia* with β-glucuronidase activity of 110,000 U/mL and sulfatase activity of 4,000 U/mL was purchased from Sigma Chemical Co. (St. Louis, MO, USA). After 16 hr, the serum mixture was loaded onto a well of a 96-well C-18 solid-phase extraction (SPE) plate (Discovery DSC-18 SPE-96 Plate, 100 mg/well; Supelco, Bellefonte, PA, USA) preconditioned sequentially with methanol (2 mL) and 5% methanol in water (2 mL). The C-18 SPE plate was then washed with 5% methanol in water (2 mL), followed by elution with 100% methanol (2 mL) to recover the steroids. The resulting methanol solution was dried under nitrogen and resuspended in 50 μL of HPLC mobile phase before liquid chromatography/tandem mass spectrometry analysis.

The HPLC system (Mitamura et al. 2000) consisted of two micropumps (both PE series 200; PerkinElmer, Norwalk, CT, USA) and an auto-sampler (PE series 200; PerkinElmer) coupled with a triple-stage quadrupole mass spectrometer (API 3000; PE-SCIEX, Concord, ON, Canada). Sample solutions were separated on a C-18 column (HyPurity Elite C18, 150 × 2.1 mm, particle size 3 μm; Thermo Hypersil, Runcorn, UK). Mobile phase A (50% methanol containing 0.5 mM ammonium formate at pH 4 at a flow rate of 200 μL/min) was used from 0 to 15 min, followed by a fast-gradient 100% mobile phase B (95% methanol containing 0.1% formic acid) within 5 min. Mobile phase B (100%) was then maintained for 4 min before a quick ramp back to 100% mobile phase A. Mobile phase A was continued for another 16 min toward the end of analysis. Target analytes were detected under a multiperiod experiment alternating between a positive mode (5,000 V) from 0 to 10 min, negative (−3,800 V) mode from 10.1 to 24.0 min, and then positive mode from 24.1 min to the end of the run. Other parameters were optimized for individual analytes. Concentrations of the metabolites were calculated using the specific peak area and corrected with the peak area of the internal standard (17α-ethynyl estradiol).

### Statistical analyses.

PCDD/PCDF and PCB values were transformed to the natural logarithm and tested for normal distribution by the Kolmogorov-Smirnov (K-S) method for parametric analyses. K-S tests for normality of the exposure data were based on the largest absolute difference between the observed and the expected cumulative distributions. Measurement values < LOD were recorded as zero in this study. Pearson correlation was used to assess the association between PCDD/PCDF levels and various estrogen metabolites. General linear regression and quadratic models were performed to evaluate the relations of body burdens of PCDDs/PCDFs and PCBs to levels of estrogen metabolites. Multivariate analyses were carried out to distinguish the independent effects on certain congeners from those of other covariable congeners. Statistical analyses were performed using the Statistical Package for Social Science (SPSS Inc. 2000), version 10.0.7 (SPSS Inc., Chicago, IL, USA).

## Results

### Subject characteristics and the levels of steroid hormones, PCDDs/PCDFs, and PCBs.

Our study subjects were pregnant without complications and had a normal birth outcome, prepregnancy body mass index (BMI) within normal range, and mean age of 28.2 or 30.4 years for those carrying a male or female baby, respectively (Table 1). Fifty-two percent of the babies were males. Only three of the women used to smoke cigarettes; none was a current smoker. Forty-eight percent of the women were passively exposed to cigarette smoke on a daily basis. However, there were no significant differences in either PCDD/PCDF exposure or serum estrogen concentrations between women who were identified as passive smokers and those who were not. None of the participants had an alcohol-consumption habit or had ever farmed or worked near an incinerator or a chemical factory (potential sources of PCDD/PCDF exposure). The mean ± SD of total PCDD/PCDF body burdens was 13.6 ± 5.1 pg WHO-TEQ/g lipid, of which 2.9 pg was due to dioxin-like PCBs.

**Figure 1:**
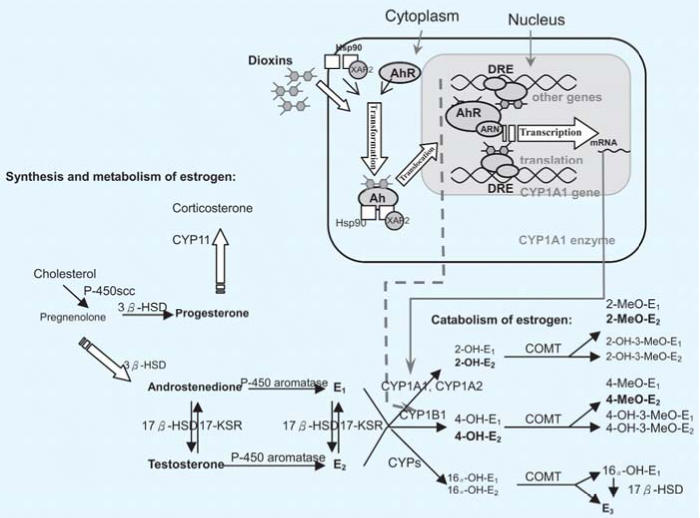
Estrogen metabolism and catabolism and potential modifications by dioxins or dioxin-like compounds. Abbreviations: 3 β-HSD, 3 β-hydroxysteroid dehydrogenase; 17-KSR, 17-ketosteroid reductase; ARNT, AhR nuclear translocator; COMT, catechol-*O*-methyl transferase; CYPs, cytochrome P450 enzymes; DRE, dioxin responsive element; XAP2, hepatitis B virus X-associated protein 2; Hsp90, 90-kDa heat-shock protein. Solid lines indicate induced CYP1A1 and CYP1A2 enzymes via AhR activation by dioxins and dioxin-like compounds; dashed lines indicate induced CYP1B1 enzyme via AhR activation by dioxins and dioxin-like compounds.

Figure 1 shows the steroid hormones quantified in the present study (in boldface) and their metabolic pathways. Table 2 shows serum levels of steroid hormones and the correlations among them. Concentrations of E_2_ and testosterone were within clinically normal ranges. Androstenedione, estrone (E_1_), estriol (E_3_), progesterone, 2-OH-E_2_, and 4-OH-E_2_ were all significantly correlated in pregnant women carrying a male fetus, with coefficients between 0.4 and 0.9. For women carrying a female fetus, E_3_ was correlated with E_1_ (*r* = 0.56, *p* < 0.01) and progesterone (*r* = 0.68, *p* < 0.001), whereas 2-OH-E_2_ was associated with 4-OH-E_2_ (*r* = 0.87, *p* < 0.001).

Table 3 shows placental levels of PCDDs, PCDFs, and PCBs in concentrations and TEQs for 50 subjects. More than 80% of the measurement outcomes were > LODs. The distributions of each level were slightly skewed to the right because geometric means were generally smaller than the middle of upper and lower limits.

### Relations of steroid hormones to levels of PCDDs/PCDFs and PCBs.

Pearson correlation results revealed a highly negative relation of 4-OH-E_2_:2-OH-E_2_ to the concentrations of TCDD, 1,2,3,7,8-pentaCDD, and total PCDD (Table 4). We also found a significantly positive relationship between the levels of high-chlorinated PCDFs, namely, 1,2,3,4,5,6,7-heptaCDF and 2-OH-E_2_. Multivariate results showed that the significant association between TCDD and 4-OH-E_2_:2-OH-E_2_ ratio (*r* = −0.111) remained significant (and also the association between 1,2,3,7,8-pentaCDD and 4-OH-E_2_:2-OH-E_2_, *r* = −0.308) after the adjustment for PCDFs, PCBs, and the mother’s age (Table 5). In addition, 1,2,3,6,7,8-heptaCDF levels were associated with increased 2-OH-E_2_ concentration after the adjustment for dioxins, PCBs, and the mother’s age.

**Figure 2:**
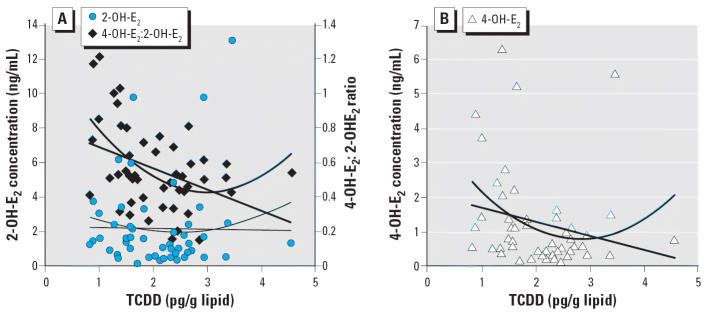
Relations of TCDD concentration to 2-OH-E_2_ concentration [*A*, left axis; −0.04(TCCD) + 2.28 (*p* = 0.93, *R*^2^ = 0.0002); quadratic model = 0.37(TCDD)^2^ − 1.8(TCDD) + 4.03 (*p* = 0.66; *R*^2^ = 0.02)], 4-OH-E_2_:2-OH-E_2_ ratio [*A*, right axis; −0.124(TCCD) + 0.82 (*p* = 0.004, *R*^2^ = 0.16); quadratic model = 0.09(TCDD)^2^ − 0.55(TCDD) + 1.25 (*p* = 0.001; *R*^2^ = 0.27)], and 4-OH-E_2_ concentrations [*B;* −0.42(TCCD) + 2.09 (*p* = 0.12, *R*^2^ = 0.05); quadratic model = 0.43(TCDD)^2^ − 2.39(TCDD) + 4.10 (*p* = 0.06; *R*^2^ = 0.12)].

The ratio of 4-OH-E_2_ to 2-OH-E_2_ decreased with increasing tertile levels of TCDD from 0.74 [95% confidence interval (CI), 0.58–0.89], to 0.48 (95% CI, 0.39–0.56), to 0.46 (95% CI, 0.37–0.55) (*p* < 0.001, β = −0.16, *R* = 0.45 by general linear regression; data not shown). Observed TCDD concentrations and levels of 2-OH-E_2_ and 4-OH-E_2_ are shown in Figure 2. Linear and quadratic models could describe the decreasing levels of 4-OH-E_2_:2-OH-E_2_ ratio and 4-OH-E_2_, respectively, with increasing TCDD. The β-value of the quadratic term is not statistically significant, and thus the linear negative association is clear. Figure 2 also shows the plot for 2-OH-E_2_ and 4-OH-E_2_ levels against TCDD concentration. 4-OH-E_2_ is negatively and significantly associated with increasing TCDD level (Figure 2B); there is no significant association between 2-OH-E_2_ and TCDD (Figure 2A). 4-OH-E_2_:2-OH-E_2_ and 2-OH-E_2_ were positively associated with 1,2,3,4,6,7,8-heptaCDF level, according to a quadratic model. However, neither achieved statistical significance (data not shown).

We also evaluated 2-MeO-E_2_ and 4-MeO-E_2_ in maternal venous serum. Only 8 of the 50 samples investigated had 2- and 4-MeO-E_2_ concentrations > LOD in serum. This could be reasonable because MeO-E_2_ might normally be efficiently excreted from the body in urine.

## Discussion

This is the first report on the association of human body burdens of PCDDs/PCDFs and PCBs with estrogen metabolites. The information presented is crucial to understand their hormonally related health effects in women, such as the risk for breast cancer. The relation between estrogen levels and exposure to PCDDs/PCDFs and PCBs has been historically difficult to investigate because of the marked variation in hormone levels during the menstrual cycle. To circumvent this difficulty, we used blood drawn at the third trimester of pregnancy, as suggested previously by Augustowska et al. (2003).

### Altered estrogen metabolism in relation to dioxins and the mechanism.

The observation of decreased E_2_ concentrations with increasing TCDD and pentaCDD levels is consistent with the known antiestrogenic properties of TCDD (Safe 2001). We have further demonstrated that decreased 4-OH-E_2_:2-OH-E_2_ ratios and decreased 4-OH-E_2_ levels are correlated with increasing TCDD level, after the adjustment of other congener exposure and maternal age. Similar results have been obtained from a study of MCF-7 and MCF-10A cells (van Duursen et al. 2003). The above observation might imply that 4-hydroxylation is a minor pathway (Figure 1) relative to 2-hydroxylation for estrogen metabolism in women with higher TCDD exposure. 4-OH-E_2_ is a strong carcinogen in comparison with 2-OH-E_2_ because 4-OH-E_2_ readily forms free radicals, such as superoxide and reactive semiquinone intermediates, via metabolic redox reactions. These free radicals may attack DNA and induce normal cells to undergo transformation into neoplastic cells (Zhu and Conney 1998). The lower 4-OH-E_2_ level, which correlated with TCDD exposure, suggests that TCDD has already modified 4-hydroxylation. This may or may not imply that TCDD reduced the carcinogenicity of estrogens. Notably, estrogen metabolism is governed by different cytochrome P450 enzymes in different types of cells. For instance, the CYP3A family is responsible for estrogen 4-hydroxylation in human liver (Kerlan et al. 1992), whereas CYP1B1 is the key 4-hydroxylation enzyme in human breast (Hayes et al. 1996) and uterus (Liehr et al. 1995). In addition, *CYP* gene expression in response to TCDD exposure may differ between cell types (Dohr et al. 1995; Kress and Greenlee 1997). The level of serum 4-OH-E_2_, which is mainly derived from hepatic metabolism, may not represent local tissue concentrations. The tissue-specific effects of TCDD on 4-OH-E_2_ productions, particularly those in extrahepatic target tissues, warrant future studies. For identification of the group at high cancer risk, present results might imply that the threshold of the cancer risk marker (4-OH-E_2_ or 4-OH-E_2_:2-OH-E_2_) might be lowered in those exposed to high levels of dioxin.

Another possibility to be considered is that TCDD exposure largely increased the capability of catechol-*O*-methyl transferase (COMT) to metabolize 4-OH-E_2_ to 4-MeO-E_2_. The significant reduction in 4-OH-E_2_ level along with TCDD exposure may then be due to a rapid subsequent metabolism and excretion of this compound. However, no biochemical evidence to date indicates that COMT uses 2-OH-E_2_ differently from 4-OH-E_2_ or that TCDD influences COMT metabolism. This deserves further investigation.

The present data highlight the complications that can arise in human studies of multi-congener exposure. For example, body burden of 1,2,3,4,6,7,8-heptaCDF was positively associated with serum levels of 2-OH-E_2_, after the adjustment for other congeners and maternal age. Further study of experimental design with single and/or multiple congener treatment would be helpful for confirming the observation. There was no significant correlation between estrogen metabolites and PCBs. This might reflect the reduced potency of PCB congeners to induce CYP1A1 and CYP1B1 (van Duursen et al. 2003).

The quadratic model reasonably described the negative association between TCDD body burden and 4-OH-E_2_ level. This might indicate that 4-OH-E_2_ tends to reach a plateau and even increase, whereas TCDD concentrations continue to increase beyond 5 pg/g lipid. This may be due to saturation of the AhR or feedback in response to decreased E_2_ level. The hypothalamus may secrete gonadotropin-releasing hormone and thus cause the ovary to produce E_2_ through the pituitary (Palter and Olive 1996).

### Other speculations and suggestions for further study.

Placental levels of PCDDs/PCDFs and PCBs in the present study were similar to those we reported previously, with 13 pg-WHO-TEQ/g lipid (Wang et al. 2004), and much lower than that reported for 21 women in Japan, with 31 pg-WHO-TEQ/g lipid (Suzuki et al. 2005). Our TEQ data and those of an earlier study of five New York women (Schecter et al. 1998) are relatively similar, although sample size and age of participants are different. The placental levels were much higher in Yu-Cheng PCB-exposed mothers (Schecter et al. 1996) than in those from Taiwan (Wang et al. 2004), Japan (Suzuki et al. 2005), and the United States (Schecter et al. 1996), as expected. More data might be necessary to draw solid conclusions on the comparisons among general populations.

The consistent observation that 2-OH-E_2_ and 4-OH-E_2_ concentrations were highly correlated is reasonable because both are metabolites of E_1_ and E_2_. This is also the case for the correlation of androstenedione with E_1_ and E_3_. Study of hormone profiles from the same subject may reduce data variation and help conclusions to be drawn, even with a limited sample size. When dividing the group according to infant sex, more correlations with estrogen metabolites appeared for women carrying male than female fetuses. This is of interest; however, when associating the estrogen metabolite levels with levels of PCDDs/PCDFs and PCBs, the sex difference was not significant.

In the present study, the total concentrations of both conjugated and free forms of estrogens were measured according to conventional methods. All women we examined showed detectable levels of 2- and 4-OH-E_2_. Many *in vitro* studies could not present such information, probably because of the efficient COMT-mediated metabolic conversion of OH-E_2_ to MeO-E_2_ (Oeltmann et al. 2004). Previous studies of cultured human mammary epithelial cells indicate that TCDD may induce expression of the *CYP1A1* and *CYP1B1* genes and catecholestrogen–mediated oxidative DNA damage (Chen et al. 2004). Epidemiologic evidence also supports a role for oxidative metabolites, particularly for catechol estrogens such as 4-OH-E_2_ (Russo et al. 2003), in initiation of breast cancer (Mitrunen and Hirvonen 2003). Thus, we suggest that OH-E_2_ rather than MeO-E_2_ should be measured directly when studying the health effects of dioxin and dioxin-like compound exposure.

### Methodologic considerations.

Possible confounders that might agonize AhR activity and alter estrogen metabolism were closely evaluated, including occupation, smoking, and cooking habits. In general, fat tissue tends to be mobilized more in pregnant than in nonpregnant women. This may provide a window for observing the interrelationships between lipophilic compounds.

## Conclusion

In conclusion, we found significantly and independently decreasing serum 4-OH-E_2_:2-OH-E_2_ ratios correlated with increasing TCDD exposure level. If TCDD is indeed a human carcinogen, then the present results imply that the congener profile should be taken into account and evaluated, in addition to genotype, to assess vulnerability and identify the groups at high risk for cancer, and that the threshold for the cancer risk marker 4-OH-E_2_:2-OH-E_2_ might be lowered in those exposed to high levels of dioxin.

## Figures and Tables

**Table 1:** General characteristics of pregnant women and their infants according to sex of the newborns.

Characteristics	Male (*n* = 26) (mean ± SD)^a^	Female (*n* = 24) (mean ± SD)^a^
Continuous variable		
Age (years)	28.2 ± 3.18	30.4 ± 3.67
Prepregnant body mass index (kg/m^2^)	22.0 ± 3.43	22.8 ± 4.24
Gestational age (weeks)	39.0 ± 1.50	38.7 ± 1.49
Baby birth weight (g)	3,251 ± 382	2,958 ± 393
Baby birth length (cm)	51.6 ± 2.59	50.9 ± 2.63
Baby head circumference (cm)	33.9 ± 1.23	33.0 ± 1.46
Placental weight (g)	629 ± 141	579 ± 184
Fat content of placenta (%)	0.74 ± 0.12	0.77 ± 0.084
Categorical variable		
Cigarette smoking, *n* (%)	1 (3.85)	2 (8.33)
Passive smoking, *n* (%)	13 (50.0)	11 (45.8)
Alcohol drinking, *n* (%)	0 (0.0)	0 (0.0)
Potential dioxin-exposed occupation, *n* (%)	0 (0.0)	0 (0.0)

No difference between sexes was statistically significant.

a Values are mean ± SD except where indicated.

**Table 2:** Mean levels of steroid hormones and estrogen metabolites and the correlations among them in serum from pregnant women at third trimester according to fetal sex.

			Pearson correlation coefficients (male, *n* = 26; female, *n* = 24)									
Hormone	GM (ng/mL)	95% CI (ng/mL)	E_1_	E_3_	Progesterone	2-OH-E_2_	4-OH-E_2_	4-OH-E_2_: 2-OH-E_2_ ratio	E_2_	Testosterone	4-OH-E_2_: E_2_ ratio	2-OH-E_2_: E_2_ ratio
Androstenedione	M 10.4	6.70–12.2	0.572**	0.663#	0.584#	0.691#	0.766#	0.356	−0.380	−0.198	0.567**	0.542*
	F 10.4	7.29–14.8	0.255	0.319	0.087	0.586**	0.556**	0.140	0.048	0.603*	0.339	0.393
E_1_	M 69.4	49.1–98.0		0.678#	0.512**	0.389*	0.440*	0.226	−0.124	0.109	0.501*	0.434
	F 59.3	41.6–84.6		0.558**	0.203	0.050	−0.153	−0.399	0.243	0.096	−0.267	−0.004
E_3_	M 367	201–611			0.800#	0.555#	0.632**	0.332	−0.097	0.064	0.561*	0.447
	F 296	206–423			0.679#	0.092	0.022	−0.111	0.220	0.229	−0.024	0.175
Progesterone	M 30.9	23.1–41.4				0.441*	0.545*	0.381	−0.171	−0.259	0.765#	0.687**
	F 34.1	25.1–46.2				−0.190	0.026	0.376	0.126	0.584*	−0.343	−0.293
2-OH-E_2_	M 1.42	0.776–2.07					0.931#	0.028	−0.259	−0.307	0.738**	0.681**
	F 1.44	0.984–2.11					0.873**	0.083	−0.348	0.032	0.774**	0.938#
4-OH-E_2_	M 0.680	0.451–1.31						0.391*	−0.177	−0.175	0.652**	0.571*
	F 0.783	0.496–1.24						0.558**	−0.382	0.097	0.930#	0.846#
4-OH-E_2_:2-OH-E_2_	M 0.479	0.412–0.556							0.121	0.285	0.050	−0.033
	F 0.544	0.435–0.680							−0.074	0.164	0.325	−0.169
E_2_	M 5.56	4.16–7.42								0.703	−0.534**	−0.661*
	F 5.15	4.10–6.48								0.216	−0.249	−0.466
Testosterone	M 9.46	6.97–12.8									−0.575	−0.603
	F 7.28	5.16–10.3									−0.417	−0.279

Abbreviations: CI, confidence interval; F, female; GM, geometric mean; M, male.

* *p* < 0.05,

** *p* < 0.01, and

# *p* < 0.001 by Pearson correlation analyses.

**Table 3:** Body burdens of polychlorinated PCDDs, PCDFs, and PCBs in concentrations and TEQs (*n* = 50).

		Concentration (pg/g lipid)		TEQ (pg-TEQ/g lipid)	
Congeners	*n*/*n*^a^	Geometric mean	95% CI	Geometric mean	95% CI
PCDDs (*n* = 7)	348/350	191	167–217	5.37	4.84–5.97
PCDFs (*n* = 10)	405/500	30.6	22.8–41.1	4.40	3.91–4.95
Non-*ortho* PCBs (*n* = 4)	162/200	30.8	25.5–37.2	1.41	1.09–1.81
Mono-*ortho* PCBs (*n* = 8)	356/400	4,330	3,660–5,130	1.38	0.347–5.50
Total TEQs	1,271/1,450	—	—	12.8	11.5–14.1
Indicator PCBs^b^ (*n* = 3)	142/150	21,300	17,100–26,800	—	—

CI, confidence interval.

a Number of detectable compounds/number of total compounds.

b The sum of PCB congeners 138, 153, 180.

**Table 4:** Correlations between body burdens of PCDDs, PCDFs, and PCBs their relations to steroid hormones in maternal venous serum.

Exposure hormone	TCDD	1,2,3,7,8-PentaCDD	Total PCDDs	1,2,3,4,6,7,8-HeptaCDF	Total PCDFs	Total WHO-TEQs	Total non-*ortho*-PCBs	Total mono-*ortho*-PCBs	Total indicator PCBs
Androstenedione (ng/mL)	−0.058	0.038	−0.035	0.175	0.062	0.022	−0.002	0.067	0.095
E_1_ (ng/mL)	−0.122	0.038	−0.091	0.257	0.085	0.016	−0.018	0.155	0.262
E_3_ (ng/mL)	0.053	0.263	0.151	0.114	0.182	0.110	−0.105	−0.063	0.013
Progesterone (ng/mL)	0.073	0.303*	0.239	0.017	0.246	0.242	0.000	0.184	0.123
2-OH-E_2_ (ng/mL)	−0.013	0.080	0.017	0.281*	0.076	0.048	0.004	0.036	0.006
4-OH-E_2_ (ng/mL)	−0.222	−0.121	−0.177	0.204	−0.115	−0.146	−0.039	−0.038	−0.043
E_2_	−0.309*	−0.313*	−0.302*	0.116	−0.258	−0.237	0.028	0.024	0.112
Testosterone	−0.099	−0.099	−0.098	−0.064	−0.075	−0.048	0.122	−0.030	0.036
4-OH-E_2_:2-OH-E_2_	−0.400**	−0.316*	−0.342*	0.078	−0.297*	−0.317*	−0.098	−0.044	0.027

* *p* < 0.05, and

** *p* < 0.01 by Pearson correlation analyses.

**Table 5:** Linear regression coefficients for predicting E_2_ metabolites by PCDDs, 0PCDFs, and PCBs.

Exposure hormone	TCDD	1,2,3,7,8-PentaCDD	1,2,3,4,6,7,8-HeptaCDF	Total non-*ortho*-PCBs	Total mono-*ortho*-PCBs	Total indicator PCBs	*R*^2^
Model group 1							
2-OH-E_2_ (ng/mL)	—	0.168	0.366*	−0.129	0.055	−0.038	0.118
4-OH-E_2_ (ng/mL)	—	−0.066	0.225	−0.118	0.052	−0.064	0.059
4-OH-E_2_:2-OH-E_2_	—	−0.308*	0.036	−0.114	−0.036	0.101	0.116
Model group 2							
2-OH-E_2_ (ng/mL)	0.161	—	0.398*	−0.167	0.054	−0.021	0.111
4-OH-E_2_ (ng/mL)	−0.160	—	0.164	−0.078	0.096	−0.108	0.073
4-OH-E_2_:2-OH-E_2_	−0.111*	—	−0.097	−0.005	0.034	0.011	0.169

TCDD and 1,2,3,7,8-pentaCDD were so highly correlated that one of them was used in either model group 1 or 2 to prevent overadjustment.

* *p* < 0.05 by multiple general linear regression analyses adjusted for other congener and maternal age.
